# Editorial: Women in cognitive neuroscience: 2023

**DOI:** 10.3389/fnhum.2025.1560661

**Published:** 2025-02-12

**Authors:** Valentina Bruno, Rania A. Mekary

**Affiliations:** ^1^Manibus Lab, Psychology Department, University of Turin, Turin, Italy; ^2^School of Pharmacy, Department of Pharmaceutical Business and Administrative Sciences, Massachusetts College of Pharmacy and Health Sciences University, Boston, MA, United States; ^3^Computational Neuroscience Outcomes Center (CNOC), Department of Neurosurgery, Brigham and Women's Hospital and Harvard Medical School, Boston, MA, United States

**Keywords:** women in STEM, cognitive neuroscience, neurodegeneration, emotional processing, attention

Despite the substantial strides made in science, technology, engineering, and mathematics (STEM), the gender gap in these fields remains a critical issue, indeed, less than the 30% of researchers worldwide are women (UIS - UNESCO Institute for Statistics, 2020). For many centuries, biases, stereotypes, and systemic barriers have impeded girls and women from pursuing careers in STEM fields. Not only is this inequitable, but also, according to UNESCO, it is important that the imbalance be addressed to foster innovation, maximize talent, enrich scientific perspectives, and achieve sustainable development (UNESCO Science Report: Towards 2030, 2021).

Cognitive neuroscience, which is dedicated to exploring the complexities of the human brain and behavior, is no exception in facing these challenges. While women have made extraordinary contributions to this domain, their work often goes underacknowledged (Fulvio et al., 2021). Amplifying their voices is crucial for inspiring the next generation of female scientists to transform traditional mindsets and redefine conventional stereotypes.

This editorial initiative highlights significant contributions by women to the field of cognitive neuroscience. The four following contributions focus on wide-ranging areas such as memory and neurodegeneration, attention, and emotional processing.

Hernández-Frausto and Vivar approach the complex connectivity between the entorhinal cortex (EC) and hippocampus (HC) regarding episodic memory formation and consolidation. They have highlighted emerging evidence pointing to a noncanonical GABAergic connection and the modulatory role of dopaminergic, cholinergic, and noradrenergic inputs for mnemonic processes. Crucially, the authors discuss in their review how early neurodegeneration in the EC-HC pathways contributes to memory dysfunction during aging and Alzheimer's disease, placing particular emphasis on the value of an important lifestyle variable, exercise, as a prophylactic intervention. Not only does such work serve to elucidate mechanisms of memory, but it also highlights the value of cross-species approaches in cognitive neurosciences. Interestingly, another study by Mitchell and Nugiel (2024) demonstrated that pubertal development and sleep disturbances significantly interacted with functional brain networks to predict mental health outcomes. Taken together, these findings underscore the need to consider physiological and developmental factors and lifestyle variables, such as exercise or sleep quality, in preserving cognitive function and preventing decline.

Simoncini et al. propose a novel psychometric tool based on spherical video technology, which is at the cutting edge in the assessment of alexithymia [i.e., difficulty in recognizing and feeling emotions (Hogeveen and Grafman, 2021)]. The integration of immersive virtual reality with psychophysiological measures overcomes the gap between implicit and explicit emotional processing. This allows for a comprehensive investigation of the ability to recognize emotions and the impairments of alexithymic individuals. The authors' work paves the way for targeted clinical interventions and exemplifies the transformative potential of emerging technologies in cognitive neuroscience.

Tosoni et al. present a mini review of the dual-network model governing visuospatial attention, focusing on the dynamic interplay between the dorsal attention network (DAN) and ventral attention network in the human brain (VAN) (Vossel et al., 2014). Their work reframes traditional models by underlining the collaborative roles of these networks in responding to attentional demands. The review discusses the distinct functions of DAN regions in attention shifting vs. maintenance, the VAN's resetting role during reorienting, and the neurophysiological underpinnings of these processes. By exploring network interactions, this research contributes to our understanding of attentional mechanisms and offers insights into the rehabilitation of spatial neglect [i.e., the inability to perceive, report and orient to sensory events toward the space contralateral to the side of the lesion (Vallar, 2001)] following brain lesions.

Montandon et al. investigated how cognitive and emotional parameters affected brain activity during perspective-taking tasks. This analytical study, by using functional magnetic resonance imaging (fMRI), has shown that fluid intelligence increases brain activity when one takes a self-perspective, while attentional deficits and a lack of inhibitory control reduce the ability to take others' perspectives. The findings also reveal that higher empathy is associated with reduced egocentric interference, while difficulties in emotion recognition are associated with amplified egocentric interference. This research gets insights in the field of social cognition, with implications for interventions at improving empathy and perspective-taking skills.

Altogether, these contributions do not only advance cognitive neuroscience, but also serve to underscore the transformative power of supporting women in research. Well-designed research addressing critical topics in neurodegeneration, emotional processing, attention, social cognition, puberty development, and mental health have showcased the various aspects of expertise and innovative approaches females bring to the field.

Looking ahead, it is important to keep forging the path that will enable gender equity to flourish in STEM, younger generations of girls to envision themselves in STEM careers, and women to break the glass ceiling. This involves mentorship opportunities, equal funding, and overcoming entrenched biases that hold women back. Institutions and journals must focus on inclusive practices, including equitable representation in key authorship roles (first and last authorships, Holman et al., 2018) and platforms to elevate underrepresented voices. It will also be of immense benefit to the scientific community for such efforts to be carried out to ensure more effective and inclusive solutions. Diversity exposes us to new perspectives, challenges deeply held paradigms, confronts outdated stereotypes, and fosters progress in ways previously unimaginable. The work in this Research Topic represents both a celebration of achievement and a call to action for further advocacy and support of women in cognitive neuroscience.
